# Phenotypic Responses, Reproduction Mode and Epigenetic Patterns under Temperature Treatments in the Alpine Plant Species *Ranunculus kuepferi* (Ranunculaceae)

**DOI:** 10.3390/biology9100315

**Published:** 2020-09-29

**Authors:** Eleni Syngelaki, Mareike Daubert, Simone Klatt, Elvira Hörandl

**Affiliations:** 1 Albrecht-von-Haller-Institute for Plant Sciences, Department of Systematics, Biodiversity and Evolution of Plants (with Herbarium), Georg-August-Universität Göttingen, 37073 Göttingen, Germany; elvira.hoerandl@biologie.uni-goettingen.de; 2 Institute of Biology and Environmental Sciences, Carl von Ossietzky University Oldenburg, 26129 Oldenburg, Germany; mareike.daubert@uni-oldenburg.de; 3 Section Safety and Environmental Protection, Georg-August-Universität Göttingen, 37073 Göttingen, Germany; simone.klatt@zvw.uni-goettingen.de

**Keywords:** abiotic stress, alpine plants, apomixis, DNA methylation, FCSS, fitness, morphological growth, polyploidy, *Ranunculus kuepferi*, temperature treatment

## Abstract

**Simple Summary:**

Environmental abiotic stimuli, e.g., temperature stress conditions, can influence the phenotype, reproduction, and epigenetics of plants. How a plant responds to cold temperature stress regarding these aspects, together with the interactions between them and the ploidy level, is still not intensively explored. Herein, we test such effects under experimental cold stress conditions on the species *Ranunculus kuepferi*, an alpine perennial herb, which mainly occurs in two cytotypes. Results suggest that diploid individuals grow better under warm conditions, while tetraploids prefer cold conditions. Moreover, phenotypic characteristics seem to correlate with the epigenetic patterns. Furthermore, cold stress conditions seem to reduce the reproductive fitness of both cytotypes. We propose that results illustrate that phenotypic plasticity, i.e., the ability of an organism, as a single genotype, to differentially respond to environmental stimuli, may affect the potential of the two cytotypes to tolerate cold stress. Finally, our study follows the geographical distribution of the species, i.e., the phenomenon that asexual taxa occupy larger geographical ranges than their sexual progenitors and extend further toward cold environments at high altitudes, which was previously observed for the natural population of the species in the European Alps.

**Abstract:**

Plant life in alpine habitats is shaped by harsh abiotic conditions and cold climates. Phenotypic variation of morphological characters and reproduction can be influenced by temperature stress. Nevertheless, little is known about the performance of different cytotypes under cold stress and how epigenetic patterns could relate to phenotypic variation. *Ranunculus kuepferi*, a perennial alpine plant, served as a model system for testing the effect of cold stress on phenotypic plasticity, reproduction mode, and epigenetic variation. Diploid and autotetraploid individuals were placed in climate growth cabinets under warm and cold conditions. Morphological traits (height, leaves and flowers) and the proportion of well-developed seeds were measured as fitness indicators, while flow cytometric seed screening (FCSS) was utilized to determine the reproduction mode. Subsequently, comparisons with patterns of methylation-sensitive amplified fragment-length polymorphisms (AFLPs) were conducted. Diploids grew better under warm conditions, while tetraploids performed better in cold treatments. Epigenetic patterns were correlated with the expressed morphological traits. Cold stress reduced the reproduction fitness but did not induce apomixis in diploids. Overall, our study underlines the potential of phenotypic plasticity for acclimation under environmental conditions and confirms the different niche preferences of cytotypes in natural populations. Results help to understand the pattern of geographical parthenogenesis in the species.

## 1. Introduction

Environment is well known to be a major factor, together with genotype, to influence the expression of a phenotype in living organisms [1]. Alpine habitats are characterized by short growth periods and cold spells, eventually with nocturnal frost during flowering time (spring or summer), which can influence several developmental processes in plants and activate adaptive traits [2,3,4]. In that context, phenotypic plasticity is defined as the ability of an organism, as a single genotype, to differentially respond to environmental stimuli [5,6]. The alterations of the phenotype can be depicted in morphology, in physiology, in gene expression, as single changes, or as a combination of these characteristics of organisms [1,7,8,9,10]. The effect of phenotypic plasticity on plants, as sessile organisms, seems to be of great importance, regarding the acclimation to environmental conditions [1,11,12]. The adaptive value of phenotypic plasticity should not be considered eminent per se, as adaptation is a complex procedure that is implied by plastic responses that are beneficial and can be transmitted to next generations [6,13,14].

As an immediate response to a change in biotic or abiotic environmental conditions, individuals often show the capacity of phenotypic accommodation [8,15], as well as acclimation to the new conditions, which are established in time [14]. The latter term is often understood as environmental tolerance, estimated through the phenotypic plasticity of several fitness traits [14,16].

In the past few decades, a growing number of studies approached phenotypic plasticity and its evolutionary aspects on organisms, as well as population level [6,10,17,18,19,20,21]. A reliable body of them highlighted the triggering role of environmental conditions and changes, which often challenge an organism’s survival and reproduction [22,23]. Studies on the genetic and epigenetic background of phenotypic plasticity [12] and its correlation with transcriptional differentiation [21,24] posed various interesting research questions on plasticity under extreme environmental conditions.

Plastic responses are the way of organisms potentially coping with extreme conditions and, thus, contributing positively to the colonization of novel habitats [25,26] via selection of fitting phenotypes [8,15,27,28,29]. Several traits could be potentially involved in such procedures [13,21]. Nicotra et al. [11] reviewed several studies on phenotypic plasticity and climate change, trying to highlight the fitness traits that seem to be up- or downregulated as part of the phenotypic response. Such phenotypic traits in plants can be stem height at maturity, flowering time and size at reproduction stage, leaf size and further morphological characteristics, and number/size of seeds [30,31,32]. For alpine plants, a reduction in stem height and dense, cushion-like growth is regarded as acclimation or even adaptation to short vegetation periods, lower temperature, and exposure to freezing [2], while polyploidization and mode of reproduction can be affected by cold stress conditions [33]. Mirouze and Paszkowski [34] proposed that this plasticity of plants to new environmental and possibly unfavorable conditions is linked to DNA methylation variation, which could regulate growth and reproduction to fit the changes [35] and may lead to microevolutionary events in plants [36].

Phenotypic plasticity can also occur in the mode of reproduction. Apomixis is defined as asexual reproduction via seeds, i.e., agamospermy [37]. It is a heritable trait [37,38] and occurs in 78 families and more than 290 genera of angiosperms [39]. Apomixis is usually facultative, which means that both sexual and apomictic seeds can be produced by the same plant, in variable proportions [37,39,40]. Apomictic plants are mostly perennials, and an individual plant may express variation in sexual/apomictic seed formation in different years [41]. Apomixis appears to be a consequence of a temporal or spatial deregulation of genes regulating the sexual pathway [42,43], but its genetic and epigenetic background and the environmental influence on the expression of that trait are rather complicated and remain enigmatic [40].

In flowering plants, apomicts are commonly polyploids. Polyploidy is thought to have many effects on vigor, physiology, morphology, and other adaptive traits [44], and genomic changes associated with the formation of polyploid cytotypes were thought to lead to the induction of apomixis [42,45,46]. However, growing evidence suggests that apomixis can originate spontaneously in diploid wild populations in low frequencies [47] and in diploid populations under cold temperature stress [41]. A successful establishment of polyploids is often connected to a niche differentiation of cytotypes [48,49]. Apomictic polyploid taxa tend to have environmental tolerances and colonization abilities at high latitudes and altitudes, which are not observed among their diploid and sexual progenitors (“geographical parthenogenesis”) [50,51,52].

Experimental research suggests that abiotic conditions like temperature and light [41,53,54,55] can directly alter the mode of reproduction in plants. Specifically, low temperature may trigger apomictic seed formation, albeit at low frequencies [41]. Case studies on apomictic clonal dandelions demonstrated the immediate response of plants to abiotic stress conditions, which are linked to methylation patterns and heritability of traits [56,57,58] and could contribute to differentiation of populations according to latitudinal gradients [59].

We hypothesized that cold exposure would influence morphological and reproductive traits; therefore, we used *Ranunculus kuepferi* Greuter & Burdet, a high-mountain perennial herb with diploids and autotetraploids as main cytotypes [60,61], as a suitable system to test our hypothesis. The reproduction mode of these cytotypes is predominantly sexual for the diploid plants and facultative apomictic for the autotetraploid plants, with varying proportions of sexual and asexual seeds [47,62]. The species is primarily distributed across the European Alps (as well as the northern Apennines and Corsica) and at altitudes between 1300 and 2800 m [47,60,61,62,63]. This distribution pattern indicates a pronounced geographical parthenogenesis pattern in the European Alps [64], where diploid populations are restricted to the southwestern Alps and tetraploid populations have colonized previously glaciated areas, i.e., northern, central, and eastern Alps [60,62,65]. Tetraploids exhibit a pronounced niche shift toward higher elevations and colder temperatures [47,63], which are associated with their reproduction mode and, in that regard, seem to provide asexual taxa with a distributional advantage [66].

Previous studies on wild populations showed that genetic differentiation between cytotypes is very low, and that within cytotypes is on a similar level (Fsts are around 0.3 for both cytotypes), independent of their reproduction mode [64]. A molecular dating revealed that the tetraploid cytotype originated only 10–80 thousand years ago [66], probably via multiple and recurrent polyploidization events [61,67]. Epigenetic studies using methylation-sensitive amplified fragment-length polymorphisms (MS-AFLPs) on the species suggested differential profiles in the cytotypes and a connection to abiotic environmental conditions of the epigenetic variation in natural populations and experimental treatments [68,69]. This epigenetic variation was further correlated with an elevation in natural populations [68] and showed higher persistence under cold treatment in experimental conditions [69]. Thus, we assume a putative epigenetic background of the niche shift of tetraploids in the Alps, which helps to understand the geographical parthenogenesis scenario for the species. Additionally, concerning several epigenetic mechanisms, it is indicated that methylation patterns also configure, together with genotype, phenotypic plasticity, under changing environmental and developmental conditions [23,70].

The aim of the current study was to investigate the putative temperature sensitivity of morphological responses under the different ploidy levels of individuals, in order to decipher how the two cytotypes cope with different temperature conditions. For that purpose, we exposed diploid and tetraploid plants of *R. kuepferi* to different controlled temperature treatments, to quantify plasticity and their phenotypic response. Furthermore, we explored the effect of treatment on the reproduction mode of the species and more specifically the probability of a positive correlation between cold temperature and induction of apomixis, as previously reported by Klatt et al. [41].

By assessing methylation variation data of the same year of treatment [69], we focused on possible correlations of phenotypic patterns with MS-AFLP profiles. We investigated whether the methylation profiles of vegetative parts differentiate for phenotypic traits according to ploidy and treatment conditions. The results provided us with insights into the potential of *R. kuepferi* to acclimate to cold conditions during the postglacial establishment of the species in the European Alps.

## 2. Materials and Methods

### 2.1. Plant Material and Experimental Design

Diploid and tetraploid individuals of the species *Ranunculus kuepferi* were collected at 102 sampling sites throughout the distribution range of the species in the European Alps [47] during the flowering seasons of 2013 and 2014. Consequently, they were transferred to the Old Botanical Garden of Göttingen University, where they were repotted in garden soil and overwintered outdoors. Their ploidy level was determined via flow cytometry measurements on silica-gel-dried leaf material collected in the field [47].

During the early spring of 2014 (beginning of sprouting and flowering period), a subset of these individuals (see Table S1, Supplementary Materials) was placed in two climate chambers MC1000E (Snijders Scientific, Tilburg, the Netherlands), which implemented different temperature conditions but all other environmental variables were kept equal [41]. The conditions of the cold and warm temperature treatments are presented in Table 1. The current experimental design was favored for the purpose of investigating the temperature preferences of the two cytotypes implied by Schinkel et al. [47] and Kirchheimer et al. [63], with cold temperature treatments simulating the harsh high alpine temperature conditions of the tetraploid cytotype’s typical habitats. The treatment of Klatt et al. [41] was kept until 2016 and rotated in 2017 before the beginning of the present study [69]. All measures were taken from 2017 under the conditions specified in Table 1.

In the early spring of 2017, 353 individuals from 63 populations were sampled, targeting as precisely as possible the distribution range of the species in the Alps (see Table S1, Supplementary Materials). The individuals were categorized into four groups regarding their treatment and ploidy: cold diploids, cold tetraploids, warm diploids, and warm tetraploids (CD, CT, WD, and WT, respectively). During the flowering period of 2017, leaf material was collected from all the plants, which was stored in silica gel for further molecular analysis.

### 2.2. Morphological Growth Variables/Seed Set/Reproductive Fitness

Flowers of the diploid individuals are self-incompatible, and they have fewer carpels and more well-developed stamens than the ones of tetraploids, which are also to some degree self-compatible [64]. Although tetraploids are mainly apomictic, pollen is still needed for endosperm formation, as it fertilizes the polar nuclei (pseudogamy) [62,64,65,71]. Hence, we placed the two cytotypes in separate space sections of the climate chambers and pollinated the individuals manually at least thrice with the pollen of plants belonging to the same ploidy level and treatment. To prevent any unwanted cross-pollination event, flowers were covered with small perforated plastic bags as long as they were blooming.

At the peak of the flowering time, measurements of the vegetative parts of the individuals (153 individuals from both growth chambers, belonging to 50 populations) were conducted during the peak of the flowering period of each chamber., regarding the height of the stem (including flower), the number and length of the leaves, and the number of flowers per individual. These morphological growth variables are most informative according to Schinkel et al. [47]. All measurements took place the same day for each chamber and were handled further in order to investigate any potential ploidy and treatment effect between the groups.

At the late stage of pollinated flowers/early stage of achene formation, stems were sealed tightly with tape in small perforated plastic bags until the harvesting of ripe fruits (single-seeded achenes). Well-developed achenes were separated from the undeveloped achenes manually by using forceps, with the former resisting the pressure because of their properly formatted endosperm and the latter shattering as they were empty [41,47]. Thereupon, the seed set, i.e., the proportion of well-developed achenes of the total number of achenes per flower per individual, was calculated, as a measure of reproductive fitness of both cytotypes under both treatments. Well-developed achenes, after being kept for at least a week at room temperature, were placed into paper bags and stored on silica gel at 4 °C, prior to further analyses.

### 2.3. Methylation-Sensitive Amplified Fragment-Length Polymorphisms (MS-AFLPs)

A subset of 100 randomly selected individuals (25 per group; Table S1, Supplementary Materials), originating from 57 populations, was selected to elucidate the effects of temperature treatments on the DNA methylation. DNA was isolated from the dried leaf material collected in 2017, using the Qiagen DNeasy Plant Mini Kit, and was processed further with a slightly modified protocol of Paun et al. [72], to investigate the patterns of epigenetic variation through the methylation-sensitive amplified fragment-length polymorphisms (MS-AFLPs). Regarding fragment scoring, the resulted electropherograms from the ABI Prism 3700/3730 (Applied Biosystems, Waltham, MA, USA) capillary sequencer went through the following scoring pipeline: Peakscanner v.2 (Applied Biosystems, Life Technologies Corporation, Carlsbad, CA, USA), RawGeno 2.0-1 [73], and MSAP_calc script [74]. A detailed wet lab and fragment scoring methodology can be found in Methods S1 (Supplementary Materials), and the DNA methylation study results on the species *Ranunculus kuepferi* are presented elsewhere [69].

### 2.4. Flow Cytometric Seed Screening (FCSS)

The flow cytometric seed screening (FCSS) method was employed to determine the reproduction mode of each seed (achene) and investigate whether temperature, especially cold stress, affects it, by quantifying the proportion of sexual versus asexual seed formation. FCSS can distinguish the ploidy levels of both endosperm and embryo per single seed and allows the reconstruction of reproductive pathways, by calculating the ratios of endosperm to embryo ploidy levels, which differs between sexual and apomictic seeds [75].

Up to 10 well-developed seeds (when applicable) per individual were analyzed with a slightly modified FCSS protocol [75], initially introduced by Schinkel et al. [47]. Seeds were prepared in separate 2 mL Eppendorf tubes with two steel beads (Qiagen, Hilden, Germany) (Ø 4 mm) for each tube and were ground with Tissue Lyzer II (Qiagen, Hilden, Germany) (stroke rate: 30 Hz for 7 s). Subsequently, nucleus isolation and staining were performed in two steps using the Otto buffers [76,77,78]. First, 200 μL of Otto I buffer was added to the ground seed material for a minimum of 30 s, to extract the nuclei from the cells. Then, 30 μm mesh filters (CellTrics, Partec GmbH, Münster, Germany) were used to filtrate the mixture into 3.5 mL plastic tubes (55 × 12 mm, Sarstedt, Nümbrecht, Germany) and the plastic tubes containing the filtrate were placed in a dark chamber to proceed with the staining of nuclei. Second, 800 μL of Otto II buffer, containing the stain 4’,6-diamidino-2-phenylindole (DAPI), in a concentration of 300 μg·mL^−1^, was added to the filtrate and, after an incubation of minimum 5 min in the dark, the measurement of the final solution was performed on a CyFlow Space flow Cytometer (Sigma-Aldrich, Partec GmbH, Münster, Germany) in the blue fluorescence channel (ultraviolet (UV) light-emitting diode (LED), wavelength 365 nm). A diploid and a tetraploid *R. kuepferi* plant were used as an external ploidy reference standard to adjust the gain of the UV LED lamp, and parameters were kept equal. Resulting histograms presenting the Gaussian means were obtained and analyzed with the FloMax software, version 2.81 (Quantum Analysis GmbH, Münster, Germany).

The Gaussian means of the peaks refer to the mean values of the DNA content for every tissue, uncovering the ploidy levels of the embryo and the endosperm of the seed. The peak index of each seed, i.e., the ratio of the mean peak value of the endosperm to the mean peak value of the embryo, was calculated, as it is required for the interpretation of plausible reproductive pathways (see Figure S1, Supplementary Materials). To link peak ratios to reproductive pathways, the studies of Cosendai and Hörandl [60], Schinkel et al. [47], and Klatt et al. [41] on *R. kuepferi* were utilized (see Table S2, Supplementary Materials). A peak index threshold of 1.65 was set to classify all seeds with lower peak indices as sexual. Seeds with peak indices values of minimum 2.0 were classified as asexual (apomictic) (see Figure S1, Supplementary Materials). The proportions of different reproduction modes of the seeds were quantified for each individual and, thereafter, were pooled for each of the four groups, in order to pairwise compare their reproductive modes.

### 2.5. Statistical Analyses

Regarding the pairwise comparisons of the Groups (WD, CD, WT, CT), multiway ANOVAs and the nonparametric Wilcoxon and Kruskal–Wallis tests were computed on morphological growth, seed set, and reproduction mode datasets. All datasets mentioned above were handled as table formats in Excel 2016, and percentages of seed set and reproduction mode (sexuality, apomixis, and BIII hybrids) were arcsine transformed to match the normal distribution of the data. Analyses were performed in R [79] under R Studio environment [80]. The visualization of descriptive statistics was carried out with ggplot2 R package [81]. All data used for descriptive statistics can be found in Table S3 (Supplementary Materials).

To compare the DNA methylation data produced previously [69] with the current morphological growth dataset, non-Euclidean, Jaccard distances of DNA methylation data were calculated under vegan 2.5-6 R package [82] and visualized with ggplot2, with color referring to the morphological growth data and shape to the predefined groups. Linear models (LMs) and generalized linear models (GLMs), produced under R, were also employed to investigate these relationships further and check the hypothesis of morphological growth data being predicted by the different types of epiloci. Group was selected as an extra predictor, as it indicated the ploidy level and the treatment conditions for each individual.

We conducted linear models for morphological traits (stem height and leaf length) which are continuous numerical variables, while for the traits that refer to count data (number of flowers and number of leaves), generalized linear models, assuming Poisson distributions, were employed [83,84]. The normal distribution of the continuous variables was tested by Shapiro–Wilk’s test, prior to linear modeling. For stem height, only the observations that differed from zero were used. As the resulting *p*-values of Shapiro–Wilk’s test were not significant, the null hypothesis, i.e., that data follow a normal distribution, could not be rejected, and we did not need to further transform those variables. Furthermore, we set the intercept, i.e., the expected mean value of *y* when all *x* = 0, to zero, regarding both types of statistical models. Without this step, the coefficients estimated the mean in each group but the difference from a reference group.

## 3. Results

### 3.1. Morphological Growth Data

**Figure 1:**
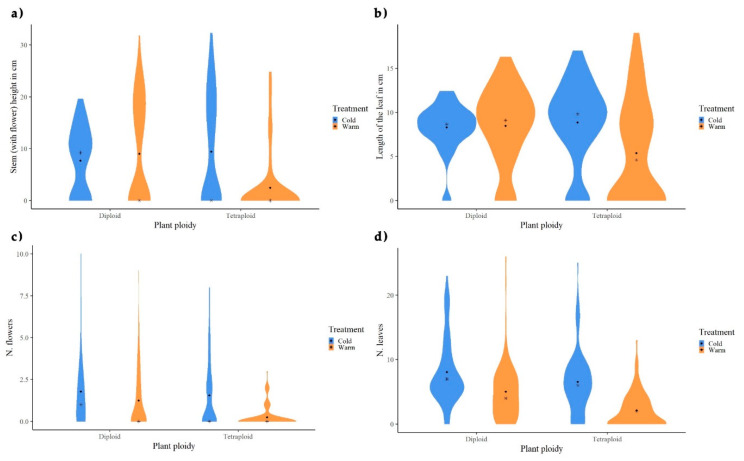
Violin plots of morphological traits of diploid and tetraploid *Ranunculus kuepferi* individuals under cold (blue) and warm (orange) temperature treatments: (**a**) stem height (with flower) in cm, (**b**) length of the longest leaf per plant in cm, (**c**) total number of flowers per plant, and (**d**) total number of leaves per plant.

Overall, the growth of tetraploid plants was affected under warm treatment, as they produced significantly lower numbers of flowers and leaves, as well as significantly shorter leaves and shorter stems in the warm treatment than in the cold one (Figure 1). Paralleling the two ploidy levels under warm treatment, we observed that diploid plants attained significantly higher values for all the morphometric measurements than the tetraploids (Figure 1). Moreover, diploid individuals developed significantly more leaves under the cold treatment than under the warm one. Herein, differences were regarded as highly significant at the 5% level of probability and slightly/marginal significant at the 10% level of probability.

### 3.2. Seed Set (Reproductive Fitness) and Reproduction Mode 

**Figure 2:**
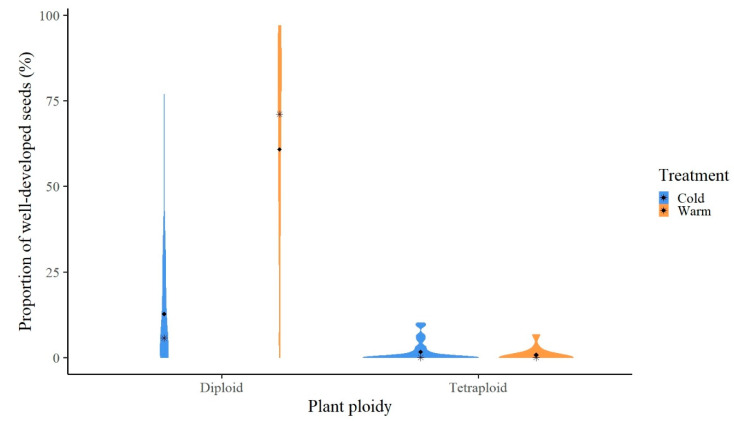
Violin plots of reproduction fitness of diploid and tetraploid *Ranunculus kuepferi* individuals under cold (blue) and warm (orange) temperature treatments.

A total number of 14,404 seeds were harvested from 143 individuals (49 populations) in both treatment conditions. On average, 20.25% of them were well developed (Figure 2), classified among CD, CT, WD, and WT with percentages of 10.97%, 4.7%, 84.13%, and 0.2%, respectively. Statistical analyses on the seed set data showed that diploids have a significantly higher seed set than tetraploids under both treatments, while diploids produced significantly more well-developed seeds under the warm treatment (Figure 2).

From the 701 FCSS measurements, 688 seeds, collected from 87 individuals, were clearly interpretable, while 13 were excluded from further analyses as there were extreme irregularities in embryo and endosperm development (histograms in Figure S1; reproductive pathways in Table S2; FCSS data in Table S3, Supplementary Materials). Flow cytometric seed screening results confirmed that the sexual mode of reproduction is dominant for the diploid individuals, whereas apomixis is higher expressed in the tetraploid cytotype (see Figure S2, Supplementary Materials). Except for sexual and apomictic seeds, in which the ploidy of the embryo is equal to that of the mother plant, there were cases detected with a ploidy shift in the embryo compared to the mother plant. In such cases, which are defined as BIII hybrids [37] (Figure S1b, Supplementary Materials), an unreduced egg cell was fertilized by reduced pollen, resulting in a higher-ploid embryo. BIII hybrids are considered to perform partial apomixis (i.e., apomeiosis only) and be a potential pathway to polyploidization [67]. Interestingly, a relatively high number of individuals (16) in the WD group displayed partially apomictic reproduction as they were classified as BIII hybrids (Figure S2).

### 3.3. Comparison of Morphological Growth Data with MS-AFLP Data 

**Figure 3:**
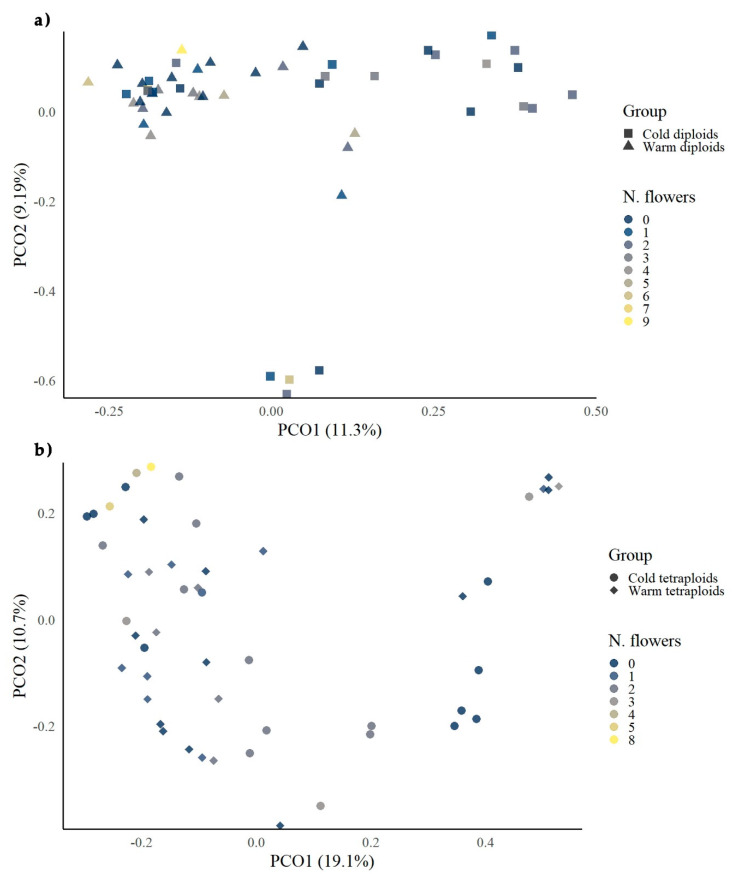
PCoAs of DNA methylation-sensitive amplified fragment-length polymorphisms (MS-AFLPs) against the number of flowers on treatment level of *Ranunculus kuepferi* diploid and tetraploid individuals: (**a**) diploid plants; (**b**) tetraploid plants.

**Figure 4:**
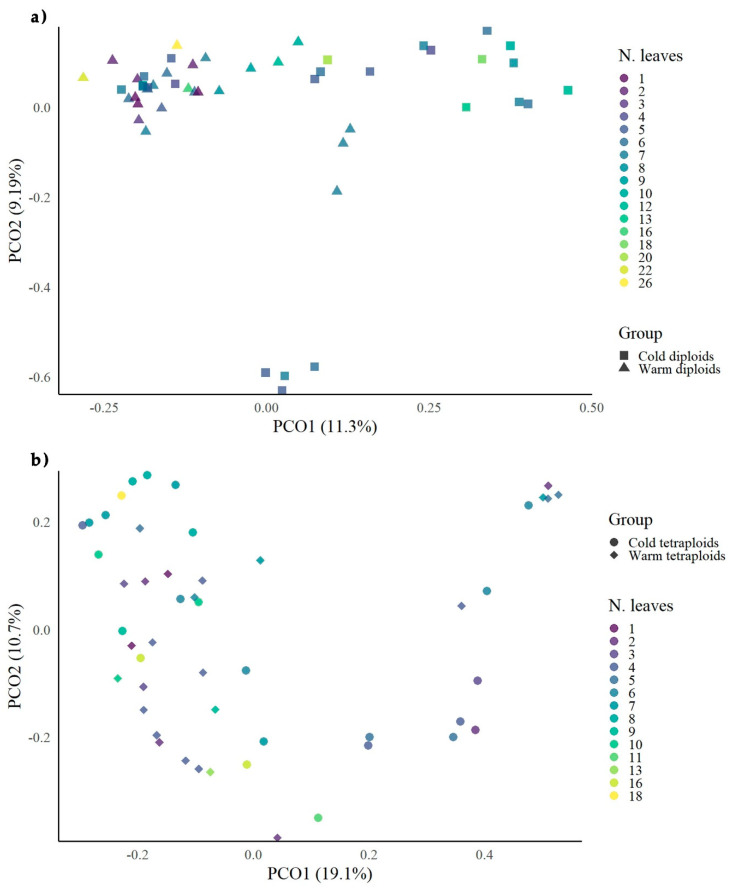
PCoAs of DNA methylation patterns (MS-AFLPs) against the number of leaves on treatment level of *Ranunculus kuepferi* diploid and tetraploid individuals: (**a**) diploid plants; (**b**) tetraploid plants.

In a previous study, a ploidy effect was confirmed for all three types of epiloci (internally, externally, and nonmethylated) under cold conditions and for externally and internally methylated under warm conditions. Moreover, a treatment effect was observed in diploids regarding the internally methylated epiloci [69]. The Jaccard distance matrices of the respective MS-AFLP data for all types of epiloci, calculated separately for each cytotype, were visualized as multidimensional scaling diagrams and present a pronounced variation of the groups and a “relaxed” correlation of the morphological traits with the epigenetic data. Treatments are more sharply separated in the diploid cytotype than in the tetraploid one, as cold diploids are gathered mainly in two clusters, whereas the epigenetic patterns of the tetraploid groups seem to overlap a lot (Figure 3 and Figure 4).

Concerning the number of flowers for each cytotype, diploids did not seem to form an epigenetic cluster by increasing or decreasing the number of flowers per individual. As already shown by Figure 1c, treatment affects this morphological trait, as the highest values were found in warm diploids, while cold diploids had mostly individuals with zero to one flower (Figure 3a). For the tetraploids, the highest numbers of flowers were found under cold conditions, and these individuals tended to cluster in MS-AFLP patterns toward the *y*-axis (Figure 3b).

The correlation of epigenetic patterns with the number of leaves was slightly stronger. MSAP patterns, regarding both treatments, tended to gather on “relaxed” clusters along the *x*-axis (toward *y*-axis) for diploid individuals with a higher number of leaves in the warm treatment (Figure 4a), while, in tetraploids, there was a slight differentiation of leaf number and epigenetic patterns along the *x*-axis, with the highest numbers of leaves in the cold treatment (Figure 4b).

Further comparisons of the two datasets via linear models (LMs) and generalized linear models (GLMs) unveiled that nonmethylated epiloci (predictor) have a significant and a slightly significant negative correlation with leaf length and number of leaves (responses), respectively (Table 2). Furthermore, modeling results showed that groups were, mostly, highly significant and positively correlated with all different morphological traits, except for the case of WT regarding the number of flowers and the externally methylated epiloci (Table 2 b). All linear models presented here (Table 2 a) had highly significant *p*-values.

## 4. Discussion

In the current study, the variability of several morphological traits together with shifts of the reproduction mode in two cytotypes of *R. kuepferi* along cold (stress) and warm (control) temperature treatments was explored, simulating natural conditions in the Alps. The results confirmed the correlation of phenotypic responses with the ploidy level and treatment conditions, while, for reproduction mode, only the ploidy effect was confirmed as reported previously [41], as there were no significant differences in treatments.

Furthermore, tetraploids had significantly improved growth under the cold treatment and seemed to suffer under the warm conditions. Comparing the growth response of the two cytotypes under warm conditions, diploids did better than tetraploids. This prominent reaction of the tetraploid cytotype to the cold treatment, in addition to the slightly better performance of the diploid cytotype under warm treatment, strengthens the hypothesis of the ecological background of the geographical parthenogenesis pattern of the species [47,66]. This hypothesis proposes the occurrence of the diploid cytotype in the warmer climate of the southwestern Alps and of the tetraploid cytotype in colder conditions at higher elevations in the rest of the Alps.

The interaction between morphological traits and DNA methylation patterns, evaluated for different cytotypes, could indicate an aspect of the molecular, more specifically, epigenetic, background of environmental response and/or phenotypic plasticity.

Overall, the treatment shift did affect the phenotypic plasticity of the two cytotypes. A repeated exposure to a condition for several flowering periods may induce acclimation and eventually put adaptive procedures in motion. Below, we attempt to disentangle and interpret all results.

### 4.1. Phenotypic Plasticity and Morphological Traits

Regarding the response of the four selected morphological traits to the treatments, our findings support the hypothesis of a niche preference depending on the cytotype documented by Kirchheimer et al. [63,66]. The observed ploidy effect under warm treatment and the treatment effect for the tetraploids under cold treatment imply a potential of phenotypic plasticity, which could sustain fitness under shifted environmental conditions [85]. In natural environments, plants show phenotypic variation under a wider range of conditions. If such a variation is inherited by next generations, it is a trait, which could be associated with adaptation and evolution [86,87,88,89]. This ability could give an advantage to an organism to colonize freshly available ecological niches. Under this process, the evolution of several adaptive traits could be expected, in favor of a successful establishment to the new niche [85,90,91]. Moreover, cytotypes with low genetic divergence [61,64] rely on phenotypic variation to manage to colonize different environments [92]. Thus, we suggest that the post-glacial colonization of the Alps by tetraploid populations of *R. kuepferi* and the distribution of the two cytotypes was fostered by their phenotypic response to climatic conditions.

Previous studies on morphological traits in natural populations of *R. kuepferi* [47] showed that tetraploid plants in the Alps often exhibit “alpine dwarfism” as an adaptation to higher elevations and harsh conditions [2]. Our results suggest that tetraploids grow best under a cold treatment, which approximates the optimal alpine conditions of the natural habitats for this cytotype, as they do significantly better than those under warm treatment. Our experiments did not include the extreme cold conditions that occur at the highest elevations of the Alps [47], which explains why we did not observe dwarf growth. Although temperature, as an environmental factor, plays the most decisive role in the ecology of each cytotype [63], the phenotypic response of the tetraploid cytotype under cold conditions may entail a spatiotemporal heterogeneity of several environmental variables, e.g., moisture, pH, and nutrients [21]. Kirchheimer et al. [63] found a niche shift of tetraploids toward more acidic soils. At the highest elevations in the Alps, nutrient-poor soils may also reduce growth performance [2]. Furthermore, we hypothesized that the adaptive effects of polyploidy, e.g., increasing cell size [44] may be expressed in a stronger way in the climate growth chambers, where only temperature varies and the other proxies are kept equal for the scope of current experimental design. For the diploids, our results are consistent with the study on natural populations [47], as diploids grew better and had more flowers than tetraploids under warm conditions. Our warm treatments appear to match their natural optimal conditions. The higher number of flowers, together with better seed set [47], resulted in a higher seed yield for diploids and, hence, a fitness advantage in their optimal, warmer climatic niche.

### 4.2. Epigenetic Patterns and Morphological Traits

The epigenetic background of phenotypic plasticity was noteworthily discussed [15,19,34,85,93], while the potential of epigenetic inheritance of plastic phenotypic traits in plants was debated by several authors [89,94,95], with cytosine (DNA) methylation being quite important in such procedures [96]. DNA methylation is an epigenetic mechanism, which seems to be strongly correlated with phenotypic plasticity to internal and external stimuli [90], which could be advantageous to occupy a wider distribution niche [22,97]. Such stress-induced epigenetic patterns provide rapid responses to fluctuating environmental conditions and could have an impact on individual fitness [22,88,98,99,100,101,102,103]. In case this phenotypic variation is heritable, DNA methylation could provide a mechanism of adaptive microevolution in plants [36,104], which is faster and independent from traditional genetic evolution [88,105,106]. Epigenetic variation differed in *R. kuepferi* between cytotypes, and also varied under climatic conditions, both in natural populations [68] and under controlled conditions [69].

Here, we compared the DNA methylation patterns with all the measured morphological traits per individual, keeping in mind their ploidy level and treatment conditions. Furthermore, we focused on the number of leaves and number of flowers, as they were important for our hypotheses regarding the survival and the fitness of *R. kuepferi*. Concerning the leaves as a morphological trait, it was intriguing to inspect their interaction with the epigenetic patterns, as they are the main photosynthetic organ of plants [107]. Tholen et al. [108] suggested that biomass production of leaves is the main factor influencing photosynthesis, while Yamori et al. [109] appraised the temperature acclimation of photosynthesis, its underlying mechanisms, and their heritable potential. Moreover, photosynthetic plasticity is triggered by stress environmental conditions in cotton cultivars [110]. In *Arabidopsis thaliana*, the phenotypic plasticity of leaves has an epigenetic basis and is associated with DNA hypomethylation [23].

The correlation of some morphological traits with the epigenetic patterns, which was indicated in our study, may refer to the invoked reaction of the individuals toward the shifted conditions after three years [69] of being acclimated in previous experimental treatments [41]. In particular, our findings showed a stronger correlation of phenotypic plasticity in leaves than in flowers with epigenetic variation, while, for both cytotypes, leaves exhibited higher values under the cold treatment.

Hence, the stress response of individuals was expressed as phenotypic plasticity, which may be controlled by DNA methylation variation, as well as by the genetic background. Such a hypothesis of epigenetic control on the phenotypic plasticity of the species was further supported by the negative correlation of the leaf length and the number of leaves with the patterns of nonmethylated epiloci for all the predefined groups. The significant relationship of nonmethylated epiloci with leaf length and the number of leaves, in comparison to the nonsignificant effect of the other types of epiloci, may highlight the importance of this epilocus in the mechanisms of phenotypic response, e.g., the gene expression toward the new temperature conditions. Nonmethylated epiloci are often linked with DNA demethylation, which is responsible for variations in phenotypic plasticity, by extending its environmental sensitivity [23], while global demethylation of genomic DNA in response to abiotic environmental stress could regulate gene expression [111,112]. However, the observed nonmethylation patterns could also reflect an underlying genetic variation and should rather be regarded as indicative of a high methylation dynamics under stress conditions [113].

The observed variation in some of the morphological traits and the epigenetic patterns seemed to affect the fitness of individuals under the new environmental conditions, thus proposing a Jack-and-master scenario [114] for the species. In that scenario, changes in traits can contribute to a higher fitness and/or be opportunistic, e.g., because of epigenetic asymmetry, profiting in such a way the establishment of a species in a new environment. Epigenetic asymmetry is often observed under changing environmental conditions, as stochastic epigenetic changes may result in high levels of plasticity, “weird” phenotypes, and even developmental disturbances [22,115,116,117,118,119].

Nevertheless, the degree of phenotypic and epigenetic response of the species in fluctuating biotic and abiotic environmental conditions is a rather complicated process, where exposure time may also play a role [22]. Thus, further studies, which would investigate the mechanisms of phenotypic response and factors that prompt it, e.g., exploring harsher environmental conditions, will help us to address our hypotheses more efficiently.

### 4.3. Reproduction Mode under Temperature Treatments

Differences in seed set and reproduction mode among the cytotypes confirmed the results of previous studies on the species in natural populations [47,60] and under experimental conditions [41]. Seed set is negatively affected by cold conditions, with diploids having significantly lower abortion rates under both temperature conditions, while tetraploids produced only a handful of well-developed seeds under warm conditions. Cold and frost conditions decrease seed set and injure the reproductive tissues of alpine plants, e.g., in *Saxifraga bryoides* [120] and *Ranunculus hirtellus* [121]. Moreover, Ladinig et al. [122] suggested that the repeated moderate frost treatment, applied also here, mimics temperature conditions occurring in high mountains and provokes frost injury in reproductive shoots, which could also result in full fruit loss. Such damaging effects were observed by Klatt et al. [41] regarding the present experimental design.

Results of the current study did not imply a significant cold-induced production of apomictic seeds in the diploid cytotype as observed by Klatt et al. [41], but rather suggest a phenotypic plasticity on reproduction mode from one year to the other in these perennial plants, as observed in the earlier study. In accordance with earlier findings [69], we speculate that these results may be correlated with the shift of treatment for the plants, which activated the complex stress responses in plants [123] such as the plastic development of flowers in *R. kuepferi*. More specifically, not all individuals produce flowers every year, as they can rest for one or more years. It is also hypothesized that female development takes place before sprouting, as in various alpine plants [2]. The severity of stress conditions also plays a role in the plants’ response and can underline the cost of plasticity in extreme environmental conditions [14,123,124]. Finally, the occurrence of BIII hybrids, which were detected in the group of WD, confirmed the hypothesis of a “female triploid bridge” as the first step to polyploidization and apomictic mode of reproduction in natural populations [67].

## 5. Conclusions

To summarize, temperature stress does affect phenotypic plasticity of morphological traits in *R. kuepferi*, with responses linked to DNA methylation patterns. In addition, the phenotypic plasticity of *R. kuepferi* most likely helps to acclimate the cytotypes to their respective climatic niches. If traits were heritable, then they would have an adaptive value and explain the geographical parthenogenesis pattern of the species in the Alps. The putative epigenetic background of phenotypic plasticity suggests that DNA methylation, in comparison to DNA mutations, provides rapid reactions of an organism to variable environmental conditions but does not necessarily ensure the stability of a phenotype. Thus, the high phenotypic variability of asexual organisms could allow for a higher or equal niche dynamic as for sexual plants. Regarding these interactions of DNA methylation and gene regulation, the next step in investigating the stress response of *R. kuepferi* would be to identify the gene expression profiles of both cytotypes under stress and controlled conditions.

## Figures and Tables

**Table 1:** Temperature treatment conditions during plant growth and seed formation.

Plant Ploidy	Cold Treatment		Warm Treatment	
	Diploid	Tetraploid	Diploid	Tetraploid
No. plants	164		189	
	74	90	92	97
Light regime (μmol·m^−2^·s^−1^, PAR)	ca. 700 *			
Photoperiod	16 h; 10 h of full light and 3 + 3 of twilight			
Temperature during the light/dark period (°C)	+7 °C day/+ 2 °C night; frost treatment; −1 °C cold shocks for three nights per week		+15 °C day/+ 10 °C night	

* Measured with a Quantum light meter (Spectrum Technologies Inc., Aurora, IL, USA) during the full light period (100% intensity) at the level of early leaf tips and first buds. Plants were rotated weekly in the cabinet to avoid effects of light and temperature gradients. PAR: Photosynthetically active radiation in 400–700 nm.

**Table 2:** Linear model and generalized linear model results, investigating the relationship of morphological traits with DNA methylation (MS-AFLP) patterns and predefined groups. MS-AFLP patterns are reported separately for different types of epiloci [70]: (**a**) linear models (model *p*-values < 0.0001); (**b**) generalized linear models.

	Nonmethylated Epiloci				Internally Methylated Epiloci				Externally Methylated Epiloci			
	EST	SE	*t*	*p*	EST	SE	*t*	*p*	EST	SE	*t*	*p*
**(a) Linear Models (LMs)**												
**Stem height**												
Epiloci’s arcsin	0.3109	0.9664	0.322	0.749	0.317	0.7598	0.417	0.678	−0.5181	0.7724	−0.671	0.505
Group WD	21.0011	2.4582	8.543	<0.0001	20.9473	2.2096	9.48	<0.0001	22.7812	2.2576	10.091	<0.0001
Group WT	14.9239	2.4573	6.073	<0.0001	15.14	1.8074	8.376	<0.0001	16.9496	2.5926	6.538	<0.0001
Group CD	11.3751	2.6498	4.293	<0.0001	11.3147	2.3252	4.866	<0.0001	13.3097	2.209	6.025	<0.0001
Group CT	18.653	2.3273	8.015	<0.0001	18.836	1.7315	10.879	<0.0001	20.5472	2.4101	8.525	<0.0001
**Leaf length**												
Epiloci’s arcsin	0.7476	0.3653	2.047	0.0435	−0.0095	0.3222	−0.029	0.977	−0.2165	0.3482	−0.622	0.535
Group WD	8.1467	0.9546	8.534	<0.0001	9.7367	0.9122	10.674	<0.0001	10.1492	0.9062	11.199	<0.0001
Group WT	8.9927	0.9476	9.49	<0.0001	10.5581	0.7544	13.996	<0.0001	11.1192	1.0916	10.186	<0.0001
Group CD	7.5579	0.9898	7.636	<0.0001	9.2389	0.9716	9.509	<0.0001	9.7112	0.9849	9.86	<0.0001
Group CT	9.4605	0.8641	10.948	<0.0001	10.8027	0.6849	15.772	<0.0001	11.371	1.0967	10.369	<0.0001
**(b) Generalized Linear Models (GLMs)**												
**No. of flowers**												
Epiloci’s arcsin	0.0019	0.1519	0.012	0.9901	−0.0077	0.1327	−0.058	0.9536	0.0973	0.1514	0.643	0.5202
Group WD	2.0764	0.4299	4.83	<0.0001	2.0971	0.4089	5.128	<0.0001	1.8705	0.4126	4.533	<0.0001
Group WT	0.836	0.3644	2.294	<0.05	0.8495	0.2705	3.14	<0.01	0.5777	0.4362	1.324	0.1854
Group CD	1.8357	0.4325	4.245	<0.0001	1.8601	0.4206	4.423	<0.0001	1.6387	0.4363	3.756	<0.001
Group CT	1.7965	0.381	4.715	<0.0001	1.8091	0.3075	5.884	<0.0001	1.5369	0.4789	3.21	<0.01
**No. of leaves**												
Epiloci’s arcsin	−0.5111	0.3085	−1.657	0.0975	−0.3444	0.287	−1.2	0.23	0.0663	0.3181	0.208	0.835
Group WD	8.4683	0.8596	9.852	<0.0001	8.1792	0.8334	9.814	<0.0001	7.2984	0.8359	8.731	<0.0001
Group WT	5.7235	0.7948	7.201	<0.0001	5.1557	0.6189	8.331	<0.0001	4.4241	0.9462	4.676	<0.0001
Group CD	8.8534	0.8903	9.945	<0.0001	8.4953	0.896	9.482	<0.0001	7.5825	0.9142	8.294	<0.0001
Group CT	8.6126	0.7854	10.966	<0.0001	8.1359	0.6513	12.493	<0.0001	7.5378	1.0138	7.436	<0.0001

EST: estimate of each coefficient; SE: standard error of the estimate; *t*: *t*-value, i.e., the coefficient divided by its standard error; *p*: *p*-value for the coefficient.

## Supplementary Materials

The following are available online at https://www.mdpi.com/2079-7737/9/10/315/s1: Table S1. List of individuals placed in the climate chambers. Table S2. Reproductive modes (a) and special cases of assumed reproduction modes (b) for diploid and tetraploid Ranunculus kuepferi plants under temperature treatments. Table S3. Vegetative growth data for the measured morphological traits, seed yield data and reproduction mode of the seeds in diploid and tetraploid Ranunculus kuepferi plants under temperature treatments (Supplementary excel file). Figure S1. Representative histograms of sexual (a), BIII hybrid (b) and apomictic seeds (c) of Ranunculus kuepferi. All histograms refer to seeds of diploid mother plants. Figure S2. Influence of the temperature treatments on the mode of reproduction in diploid and tetraploid Ranunculus kuepferi plants. Methods S1. Methylation-sensitive amplified fragment length polymorphisms (MS-AFLPs or MSAPs): Lab protocol and fragments scoring pipeline.
