# Supplementary material for: Phenotypic Responses, Reproduction Mode and Epigenetic Patterns under Temperature Treatments in the Alpine Plant Species Ranunculus kuepferi (Ranunculaceae)
Source: Biology (Basel). 2020 Sep 29;9(10):315. doi: 10.3390/biology9100315 (PMC7600421; doi:10.3390/biology9100315)
Supplement: Supplementary file 1 [file biology-09-00315-s001.zip › Supplementary_data/Supplementary_data.docx]

Article

Phenotypic responses, reproduction mode and epigenetic patterns under temperature treatments in the alpine species *Ranunculus kuepferi* (Ranunculaceae)

Eleni Syngelaki, Mareike Daubert, Simone Klatt and Elvira Hörandl

The following supplementary material is available for this article:

**Table S1.** List of individuals placed in the climate chambers. Listed are the sampling ID, ploidy level, treatment, country, province and altitude. The individuals, which were used for the MSAP laboratory work, are highlighted with green [42, 48, 70].

| SampleID | Leaf ploidy | Temperature treatment 2017 | Country | Region | Altitude | MSAPs |
| --- | --- | --- | --- | --- | --- | --- |
| 3_2_3 | diploid | warm | France | Provence-Alpes-Côte d'Azur | 2291 | Y |
| 3_4_2 | diploid | warm | France | Provence-Alpes-Côte d'Azur | 2291 | N |
| 3_4_3 | diploid | warm | France | Provence-Alpes-Côte d'Azur | 2291 | N |
| 23_1_2 | diploid | warm | France | Provence-Alpes-Côte d'Azur | 1616 | N |
| 23_2_2 | diploid | warm | France | Provence-Alpes-Côte d'Azur | 1616 | N |
| 23_2_3 | diploid | warm | France | Provence-Alpes-Côte d'Azur | 1616 | N |
| 23_4_1 | diploid | warm | France | Provence-Alpes-Côte d'Azur | 1616 | Y |
| 23_4_3 | diploid | warm | France | Provence-Alpes-Côte d'Azur | 1616 | N |
| 24_2_2 | diploid | warm | France | Provence-Alpes-Côte d'Azur | 1925 | N |
| 24_2_3 | diploid | warm | France | Provence-Alpes-Côte d'Azur | 1925 | N |
| 24_3_2 | diploid | warm | France | Provence-Alpes-Côte d'Azur | 1925 | N |
| 24_4_1 | diploid | warm | France | Provence-Alpes-Côte d'Azur | 1925 | N |
| 24_4_3 | diploid | warm | France | Provence-Alpes-Côte d'Azur | 1925 | Y |
| 25_1_2 | diploid | warm | France | Provence-Alpes-Côte d'Azur | 1435 | N |
| 25_2_2 | diploid | warm | France | Provence-Alpes-Côte d'Azur | 1435 | N |
| 25_2_3 | diploid | warm | France | Provence-Alpes-Côte d'Azur | 1435 | N |
| 25_3_2 | diploid | warm | France | Provence-Alpes-Côte d'Azur | 1435 | Y |
| 25_3_3 | diploid | warm | France | Provence-Alpes-Côte d'Azur | 1435 | N |
| 25_4_1 | diploid | warm | France | Provence-Alpes-Côte d'Azur | 1435 | N |
| 26_1_2 | diploid | warm | France | Provence-Alpes-Côte d'Azur | 1456 | N |
| 26_2_1 | diploid | warm | France | Provence-Alpes-Côte d'Azur | 1456 | N |
| 26_2_2 | diploid | warm | France | Provence-Alpes-Côte d'Azur | 1456 | N |
| 26_3_2 | diploid | warm | France | Provence-Alpes-Côte d'Azur | 1456 | N |
| 26_3_3 | diploid | warm | France | Provence-Alpes-Côte d'Azur | 1456 | Y |
| 26_4_3 | diploid | warm | France | Provence-Alpes-Côte d'Azur | 1456 | N |
| 27_1_1 | diploid | warm | France | Rhônes-Alpes | 1449 | N |
| 27_1_2 | diploid | warm | France | Rhônes-Alpes | 1449 | N |
| 27_2_1 | diploid | warm | France | Rhônes-Alpes | 1449 | Y |
| 27_2_2 | diploid | warm | France | Rhônes-Alpes | 1449 | N |
| 27_2_3 | diploid | warm | France | Rhônes-Alpes | 1449 | N |
| 27_3_1 | diploid | warm | France | Rhônes-Alpes | 1449 | N |
| 27_4_3 | diploid | warm | France | Rhônes-Alpes | 1449 | N |
| 28_2_2 | diploid | warm | Italy | Piemonte | 1685 | N |
| 28_3_1 | diploid | warm | Italy | Piemonte | 1685 | Y |
| 28_3_2 | diploid | warm | Italy | Piemonte | 1685 | Y |
| 28_4_2 | diploid | warm | Italy | Piemonte | 1685 | N |
| 28_4_3 | diploid | warm | Italy | Piemonte | 1685 | N |
| 29_1_1 | diploid | warm | Italy | Piemonte | 2020 | N |
| 29_1_2 | diploid | warm | Italy | Piemonte | 2020 | Y |
| 29_2_2 | diploid | warm | Italy | Piemonte | 2020 | N |
| 29_4_1 | diploid | warm | Italy | Piemonte | 2020 | N |
| 30_1_1 | diploid | warm | Italy | Piemonte | 1743 | N |
| 30_1_3 | diploid | warm | Italy | Piemonte | 1743 | Y |
| 31_3_3 | diploid | warm | Italy | Piemonte | 1937 | Y |
| 31_4_1 | diploid | warm | Italy | Piemonte | 1937 | N |
| 31_4_2 | diploid | warm | Italy | Piemonte | 1937 | N |
| 32_1_3 | diploid | warm | Italy | Piemonte | 2320 | N |
| 32_4_1 | diploid | warm | Italy | Piemonte | 2320 | N |
| 32_4_2 | diploid | warm | Italy | Piemonte | 2320 | Y |
| 33_3_2 | diploid | warm | Italy | Piemonte | 2328 | N |
| 33_3_3 | diploid | warm | Italy | Piemonte | 2328 | Y |
| 33_4_3 | diploid | warm | Italy | Piemonte | 2328 | N |
| 112_1_3 | diploid | warm | France | Provence-Alpes-Côte d'Azur | 1626 | N |
| 112_2_2 | diploid | warm | France | Provence-Alpes-Côte d'Azur | 1626 | N |
| 112_2_3 | diploid | warm | France | Provence-Alpes-Côte d'Azur | 1626 | N |
| 112_3_1 | diploid | warm | France | Provence-Alpes-Côte d'Azur | 1626 | Y |
| 112_3_2 | diploid | warm | France | Provence-Alpes-Côte d'Azur | 1626 | Y |
| 112_3_3 | diploid | warm | France | Provence-Alpes-Côte d'Azur | 1626 | N |
| 115_1_1 | diploid | warm | France | Provence-Alpes-Côte d'Azur | 1891 | N |
| 115_1_2 | diploid | warm | France | Provence-Alpes-Côte d'Azur | 1891 | N |
| 115_2_2 | diploid | warm | France | Provence-Alpes-Côte d'Azur | 1891 | N |
| 115_3_3 | diploid | warm | France | Provence-Alpes-Côte d'Azur | 1891 | Y |
| 115_4_3 | diploid | warm | France | Provence-Alpes-Côte d'Azur | 1891 | N |
| 116_1_1 | diploid | warm | France | Provence-Alpes-Côte d'Azur | 1953 | Y |
| 116_1_2 | diploid | warm | France | Provence-Alpes-Côte d'Azur | 1953 | N |
| 116_2_1 | diploid | warm | France | Provence-Alpes-Côte d'Azur | 1953 | N |
| 116_3_3 | diploid | warm | France | Provence-Alpes-Côte d'Azur | 1953 | Y |
| 117_1_2 | diploid | warm | France | Provence-Alpes-Côte d'Azur | 1632 | N |
| 117_1_3 | diploid | warm | France | Provence-Alpes-Côte d'Azur | 1632 | N |
| 117_2_1 | diploid | warm | France | Provence-Alpes-Côte d'Azur | 1632 | N |
| 117_3_2 | diploid | warm | France | Provence-Alpes-Côte d'Azur | 1632 | Y |
| 117_4_1 | diploid | warm | France | Provence-Alpes-Côte d'Azur | 1632 | N |
| 117_4_2 | diploid | warm | France | Provence-Alpes-Côte d'Azur | 1632 | N |
| 202_1_1 | diploid | warm | France | Provence-Alpes-Côte d'Azur | 1829 | N |
| 202_1_2 | diploid | warm | France | Provence-Alpes-Côte d'Azur | 1829 | N |
| 202_2_1 | diploid | warm | France | Provence-Alpes-Côte d'Azur | 1829 | N |
| 202_4_3 | diploid | warm | France | Provence-Alpes-Côte d'Azur | 1829 | N |
| 203_2_3 | diploid | warm | France | Provence-Alpes-Côte d'Azur | 1840 | N |
| 203_4_2 | diploid | warm | France | Provence-Alpes-Côte d'Azur | 1840 | N |
| 203_4_3 | diploid | warm | France | Provence-Alpes-Côte d'Azur | 1840 | Y |
| 233_1_1 | diploid | warm | France | Provence-Alpes-Côte d'Azur | 2185 | N |
| 233_2_1 | diploid | warm | France | Provence-Alpes-Côte d'Azur | 2185 | Y |
| 233_2_2 | diploid | warm | France | Provence-Alpes-Côte d'Azur | 2185 | N |
| 233_3_2 | diploid | warm | France | Provence-Alpes-Côte d'Azur | 2185 | N |
| 28B_2_1 | diploid | warm | Italy | Piemonte | 1685 | N |
| 28B_3_1 | diploid | warm | Italy | Piemonte | 1685 | Y |
| 28B_4_3 | diploid | warm | Italy | Piemonte | 1685 | N |
| 31B_1_1 | diploid | warm | Italy | Piemonte | 1937 | N |
| 31B_1_3 | diploid | warm | Italy | Piemonte | 1937 | Y |
| 31B/32B_2_2 | diploid | warm | Italy | Piemonte | 1937 | Y |
| 32B_1_2 | diploid | warm | Italy | Piemonte | 2320 | Y |
| 32B/31B_4_3 | diploid | warm | Italy | Piemonte | 1937 | N |
| 16_4_1 | tetraploid | warm | France | Provence-Alpes-Côte d'Azur | 2078 | N |
| 17_1_1 | tetraploid | warm | France | Provence-Alpes-Côte d'Azur | 2357 | N |
| 17_2_1 | tetraploid | warm | France | Provence-Alpes-Côte d'Azur | 2357 | N |
| 17_3_3 | tetraploid | warm | France | Provence-Alpes-Côte d'Azur | 2357 | N |
| 17_4_3 | tetraploid | warm | France | Provence-Alpes-Côte d'Azur | 2357 | Y |
| 34_2_1 | tetraploid | warm | France | Rhônes-Alpes | 2120 | Y |
| 34_2_3 | tetraploid | warm | France | Rhônes-Alpes | 2120 | N |
| 34_4_1 | tetraploid | warm | France | Rhônes-Alpes | 2120 | N |
| 36_1_1 | tetraploid | warm | France | Rhônes-Alpes | 2152 | N |
| 36_2_3 | tetraploid | warm | France | Rhônes-Alpes | 2152 | N |
| 36_4_1 | tetraploid | warm | France | Rhônes-Alpes | 2152 | Y |
| 36_4_2 | tetraploid | warm | France | Rhônes-Alpes | 2152 | N |
| 37_1_2 | tetraploid | warm | Italy | Val d'Aosta | 2115 | Y |
| 37_2_3 | tetraploid | warm | Italy | Val d'Aosta | 2115 | N |
| 37_3_1 | tetraploid | warm | Italy | Val d'Aosta | 2115 | N |
| 38_1_2 | tetraploid | warm | France | Rhônes-Alpes | 2182 | Y |
| 40_1_1 | tetraploid | warm | Switzerland | Wallis | 1860 | N |
| 40_2_1 | tetraploid | warm | Switzerland | Wallis | 1860 | N |
| 40_3_3 | tetraploid | warm | Switzerland | Wallis | 1860 | N |
| 40_4_3 | tetraploid | warm | Switzerland | Wallis | 1860 | N |
| 41_4_3 | tetraploid | warm | Italy | Val d'Aosta | 2174 | Y |
| 42_1_2 | tetraploid | warm | Switzerland | Wallis | 1789 | Y |
| 42_1_3 | tetraploid | warm | Switzerland | Wallis | 1789 | N |
| 42_2_2 | tetraploid | warm | Switzerland | Wallis | 1789 | N |
| 42_3_3 | tetraploid | warm | Switzerland | Wallis | 1789 | N |
| 43_4_1 | tetraploid | warm | Switzerland | Wallis | 2012 | N |
| 43_4_3 | tetraploid | warm | Switzerland | Wallis | 2012 | N |
| 45_1_3 | tetraploid | warm | Switzerland | Wallis | 2400 | N |
| 45_2_2 | tetraploid | warm | Switzerland | Wallis | 2400 | N |
| 45_2_3 | tetraploid | warm | Switzerland | Wallis | 2400 | N |
| 45_3_2 | tetraploid | warm | Switzerland | Wallis | 2400 | N |
| 45_3_3 | tetraploid | warm | Switzerland | Wallis | 2400 | N |
| 47_1_1 | tetraploid | warm | Switzerland | Graubünden | 2211 | N |
| 47_2_1 | tetraploid | warm | Switzerland | Graubünden | 2211 | N |
| 47_3_2 | tetraploid | warm | Switzerland | Graubünden | 2211 | N |
| 47_3_3 | tetraploid | warm | Switzerland | Graubünden | 2211 | Y |
| 48_1_1 | tetraploid | warm | Switzerland | Graubünden | 2262 | N |
| 48_2_2 | tetraploid | warm | Switzerland | Graubünden | 2262 | N |
| 50_1_2 | tetraploid | warm | Switzerland | Graubünden | 2322 | N |
| 50_1_3 | tetraploid | warm | Switzerland | Graubünden | 2322 | N |
| 50_3_2 | tetraploid | warm | Switzerland | Graubünden | 2322 | N |
| 50_3_3 | tetraploid | warm | Switzerland | Graubünden | 2322 | N |
| 51_3_1 | tetraploid | warm | Austria | Tirol | 2286 | Y |
| 51_3_3 | tetraploid | warm | Austria | Tirol | 2286 | N |
| 53_1_2 | tetraploid | warm | Switzerland | Graubünden | 2456 | N |
| 53_1_3 | tetraploid | warm | Switzerland | Graubünden | 2456 | Y |
| 53_2_1 | tetraploid | warm | Switzerland | Graubünden | 2456 | N |
| 53_3_2 | tetraploid | warm | Switzerland | Graubünden | 2456 | N |
| 53_4_3 | tetraploid | warm | Switzerland | Graubünden | 2456 | N |
| 54_1_1 | tetraploid | warm | Italy | Lombardia | 2303 | N |
| 54_2_3 | tetraploid | warm | Italy | Lombardia | 2303 | Y |
| 54_3_1 | tetraploid | warm | Italy | Lombardia | 2303 | N |
| 54_4_1 | tetraploid | warm | Italy | Lombardia | 2303 | N |
| 58_1_1 | tetraploid | warm | Italy | Trentino Alto Adige/ Südtirol | 2117 | Y |
| 58_3_3 | tetraploid | warm | Italy | Trentino Alto Adige/ Südtirol | 2117 | Y |
| 58_4_1 | tetraploid | warm | Italy | Trentino Alto Adige/ Südtirol | 2117 | N |
| 66_1_3 | tetraploid | warm | Italy | Trentino Alto Adige/ Südtirol | 2101 | N |
| 66_2_1 | tetraploid | warm | Italy | Trentino Alto Adige/ Südtirol | 2101 | N |
| 66_3_2 | tetraploid | warm | Italy | Trentino Alto Adige/ Südtirol | 2101 | N |
| 66_3_3 | tetraploid | warm | Italy | Trentino Alto Adige/ Südtirol | 2101 | N |
| 66_4_2 | tetraploid | warm | Italy | Trentino Alto Adige/ Südtirol | 2101 | N |
| 74_1_1 | tetraploid | warm | Austria | Osttirol | 2117 | N |
| 74_1_2 | tetraploid | warm | Austria | Osttirol | 2117 | Y |
| 74_2_1 | tetraploid | warm | Austria | Osttirol | 2117 | N |
| 75_3_1 | tetraploid | warm | Switzerland | Graubünden | 2678 | N |
| 78_3_2 | tetraploid | warm | Switzerland | Wallis | 2000 | Y |
| 79_1_3 | tetraploid | warm | Switzerland | Graubünden | 2280 | N |
| 79_2_1 | tetraploid | warm | Switzerland | Graubünden | 2280 | N |
| 79_2_3 | tetraploid | warm | Switzerland | Graubünden | 2280 | Y |
| 79_3_2 | tetraploid | warm | Switzerland | Graubünden | 2280 | N |
| 79_4_1 | tetraploid | warm | Switzerland | Graubünden | 2280 | N |
| 85_2_1 | tetraploid | warm | Austria | Kärnten | 2184 | N |
| 85_4_1 | tetraploid | warm | Austria | Kärnten | 2184 | Y |
| 88_3_1 | tetraploid | warm | Switzerland | Graubünden | 2300 | Y |
| 89_1_2 | tetraploid | warm | Switzerland | Graubünden | 2265 | N |
| 89_2_1 | tetraploid | warm | Switzerland | Graubünden | 2265 | N |
| 92_2_2 | tetraploid | warm | Switzerland | Graubünden | 2260 | N |
| 92_3_2 | tetraploid | warm | Switzerland | Graubünden | 2260 | N |
| 92_4_2 | tetraploid | warm | Switzerland | Graubünden | 2260 | N |
| 93_2_2 | tetraploid | warm | Switzerland | Wallis | 2405 | N |
| 96_1_1 | tetraploid | warm | France | Provence-Alpes-Côte d'Azur | 2300 | N |
| 96_3_1 | tetraploid | warm | France | Provence-Alpes-Côte d'Azur | 2300 | N |
| 96_3_3 | tetraploid | warm | France | Provence-Alpes-Côte d'Azur | 2300 | N |
| 103_4_1 | tetraploid | warm | Italy | Lombardia | 2290 | N |
| 103_4_2 | tetraploid | warm | Italy | Lombardia | 2290 | Y |
| 106_1_2 | tetraploid | warm | Italy | Trentino Alto Adige/ Südtirol | 2142 | N |
| 106_3_2 | tetraploid | warm | Italy | Trentino Alto Adige/ Südtirol | 2142 | N |
| 106_3_3 | tetraploid | warm | Italy | Trentino Alto Adige/ Südtirol | 2142 | N |
| 106_4_1 | tetraploid | warm | Italy | Trentino Alto Adige/ Südtirol | 2142 | Y |
| 108_4_3 | tetraploid | warm | Switzerland | Graubünden | 2171 | N |
| 111_1_3 | tetraploid | warm | France | Provence-Alpes-Côte d'Azur | 2243 | N |
| 111_3_2 | tetraploid | warm | France | Provence-Alpes-Côte d'Azur | 2243 | Y |
| 111_3_3 | tetraploid | warm | France | Provence-Alpes-Côte d'Azur | 2243 | Y |
| 116_4_1 | tetraploid | warm | France | Provence-Alpes-Côte d'Azur | 1953 | Y |
| 116_4_2 | tetraploid | warm | France | Provence-Alpes-Côte d'Azur | 1953 | N |
| 208_2_1 | tetraploid | warm | France | Provence-Alpes-Côte d'Azur | 1924 | Y |
| 3B_1_2 | tetraploid | warm | France | Provence-Alpes-Côte d'Azur | 2291 | Y |
| 3_1_2 | diploid | cold | France | Provence-Alpes-Côte d'Azur | 2291 | N |
| 3_1_3 | diploid | cold | France | Provence-Alpes-Côte d'Azur | 2291 | N |
| 3_2_2 | diploid | cold | France | Provence-Alpes-Côte d'Azur | 2291 | Y |
| 3_3_2 | diploid | cold | France | Provence-Alpes-Côte d'Azur | 2291 | Y |
| 3_4_1 | diploid | cold | France | Provence-Alpes-Côte d'Azur | 2291 | N |
| 23_3_2 | diploid | cold | France | Provence-Alpes-Côte d'Azur | 1616 | N |
| 23_3_3 | diploid | cold | France | Provence-Alpes-Côte d'Azur | 1616 | Y |
| 23_4_2 | diploid | cold | France | Provence-Alpes-Côte d'Azur | 1616 | N |
| 24_1_2 | diploid | cold | France | Provence-Alpes-Côte d'Azur | 1925 | Y |
| 24_3_3 | diploid | cold | France | Provence-Alpes-Côte d'Azur | 1925 | N |
| 24_4_2 | diploid | cold | France | Provence-Alpes-Côte d'Azur | 1925 | N |
| 25_1_3 | diploid | cold | France | Provence-Alpes-Côte d'Azur | 1435 | Y |
| 25_2_1 | diploid | cold | France | Provence-Alpes-Côte d'Azur | 1435 | N |
| 25_3_1 | diploid | cold | France | Provence-Alpes-Côte d'Azur | 1435 | N |
| 25_4_2 | diploid | cold | France | Provence-Alpes-Côte d'Azur | 1435 | N |
| 25_4_3 | diploid | cold | France | Provence-Alpes-Côte d'Azur | 1435 | N |
| 26_1_3 | diploid | cold | France | Provence-Alpes-Côte d'Azur | 1456 | N |
| 26_4_1 | diploid | cold | France | Provence-Alpes-Côte d'Azur | 1456 | Y |
| 27_2_2 | diploid | cold | France | Rhônes-Alpes | 1449 | Y |
| 27_3_2 | diploid | cold | France | Rhônes-Alpes | 1449 | N |
| 27_3_3 | diploid | cold | France | Rhônes-Alpes | 1449 | N |
| 27_4_1 | diploid | cold | France | Rhônes-Alpes | 1449 | N |
| 28_1_2 | diploid | cold | Italy | Piemonte | 1685 | N |
| 28_2_1 | diploid | cold | Italy | Piemonte | 1685 | N |
| 28_2_3 | diploid | cold | Italy | Piemonte | 1685 | Y |
| 28_3_3 | diploid | cold | Italy | Piemonte | 1685 | N |
| 29_1_3 | diploid | cold | Italy | Piemonte | 2020 | N |
| 29_2_3 | diploid | cold | Italy | Piemonte | 2020 | N |
| 29_3_1 | diploid | cold | Italy | Piemonte | 2020 | N |
| 29_4_2 | diploid | cold | Italy | Piemonte | 2020 | Y |
| 30_1_2 | diploid | cold | Italy | Piemonte | 1743 | Y |
| 31_2_3 | diploid | cold | Italy | Piemonte | 1937 | Y |
| 32_1_2 | diploid | cold | Italy | Piemonte | 2320 | N |
| 32_3_2 | diploid | cold | Italy | Piemonte | 2320 | N |
| 32_3_3 | diploid | cold | Italy | Piemonte | 2320 | Y |
| 32_4_3 | diploid | cold | Italy | Piemonte | 2320 | N |
| 33_1_1 | diploid | cold | Italy | Piemonte | 2328 | N |
| 33_3_1 | diploid | cold | Italy | Piemonte | 2328 | N |
| 33_4_1 | diploid | cold | Italy | Piemonte | 2328 | N |
| 33_4_2 | diploid | cold | Italy | Piemonte | 2328 | Y |
| 112_1_1 | diploid | cold | France | Provence-Alpes-Côte d'Azur | 1626 | N |
| 112_1_2 | diploid | cold | France | Provence-Alpes-Côte d'Azur | 1626 | Y |
| 112_2_1 | diploid | cold | France | Provence-Alpes-Côte d'Azur | 1626 | N |
| 112_4_3 | diploid | cold | France | Provence-Alpes-Côte d'Azur | 1626 | N |
| 115_1_3 | diploid | cold | France | Provence-Alpes-Côte d'Azur | 1891 | N |
| 115_2_3 | diploid | cold | France | Provence-Alpes-Côte d'Azur | 1891 | N |
| 115_3_1 | diploid | cold | France | Provence-Alpes-Côte d'Azur | 1891 | N |
| 115_4_1 | diploid | cold | France | Provence-Alpes-Côte d'Azur | 1891 | N |
| 115_4_2 | diploid | cold | France | Provence-Alpes-Côte d'Azur | 1891 | Y |
| 116_1_3 | diploid | cold | France | Provence-Alpes-Côte d'Azur | 1953 | Y |
| 116_2_2 | diploid | cold | France | Provence-Alpes-Côte d'Azur | 1953 | N |
| 116_2_3 | diploid | cold | France | Provence-Alpes-Côte d'Azur | 1953 | N |
| 116_3_1 | diploid | cold | France | Provence-Alpes-Côte d'Azur | 1953 | Y |
| 116_3_2 | diploid | cold | France | Provence-Alpes-Côte d'Azur | 1953 | N |
| 117_1_1 | diploid | cold | France | Provence-Alpes-Côte d'Azur | 1632 | N |
| 117_2_2 | diploid | cold | France | Provence-Alpes-Côte d'Azur | 1632 | N |
| 117_2_3 | diploid | cold | France | Provence-Alpes-Côte d'Azur | 1632 | N |
| 117_3_3 | diploid | cold | France | Provence-Alpes-Côte d'Azur | 1632 | Y |
| 202_1_3 | diploid | cold | France | Provence-Alpes-Côte d'Azur | 1829 | N |
| 202_3_3 | diploid | cold | France | Provence-Alpes-Côte d'Azur | 1829 | Y |
| 203_2_2 | diploid | cold | France | Provence-Alpes-Côte d'Azur | 1840 | Y |
| 203_4_1 | diploid | cold | France | Provence-Alpes-Côte d'Azur | 1840 | N |
| 233_2_3 | diploid | cold | France | Provence-Alpes-Côte d'Azur | 2185 | Y |
| 233_3_1 | diploid | cold | France | Provence-Alpes-Côte d'Azur | 2185 | N |
| 233_3_3 | diploid | cold | France | Provence-Alpes-Côte d'Azur | 2185 | N |
| 28B_1_1 | diploid | cold | Italy | Piemonte | 1685 | N |
| 28B_1_2 | diploid | cold | Italy | Piemonte | 1685 | Y |
| 28B_1_3 | diploid | cold | Italy | Piemonte | 1685 | N |
| 28B_2_2 | diploid | cold | Italy | Piemonte | 1685 | N |
| 31B_3_3 | diploid | cold | Italy | Piemonte | 1937 | Y |
| 32B_1_1 | diploid | cold | Italy | Piemonte | 2320 | Y |
| 32B/31B_2_1 | diploid | cold | Italy | Piemonte | 2320 | Y |
| 32B_2_3 | diploid | cold | Italy | Piemonte | 2320 | N |
| 32B_3_2 | diploid | cold | Italy | Piemonte | 2320 | N |
| 17_2_3 | tetraploid | cold | France | Provence-Alpes-Côte d'Azur | 2357 | N |
| 17_3_1 | tetraploid | cold | France | Provence-Alpes-Côte d'Azur | 2357 | N |
| 17_3_2 | tetraploid | cold | France | Provence-Alpes-Côte d'Azur | 2357 | N |
| 17_4_1 | tetraploid | cold | France | Provence-Alpes-Côte d'Azur | 2357 | N |
| 17_4_2 | tetraploid | cold | France | Provence-Alpes-Côte d'Azur | 2357 | Y |
| 34_1_1 | tetraploid | cold | France | Rhônes-Alpes | 2120 | Y |
| 34_2_2 | tetraploid | cold | France | Rhônes-Alpes | 2120 | N |
| 36_1_2 | tetraploid | cold | France | Rhônes-Alpes | 2152 | N |
| 36_1_3 | tetraploid | cold | France | Rhônes-Alpes | 2152 | N |
| 36_2_1 | tetraploid | cold | France | Rhônes-Alpes | 2152 | Y |
| 36_2_2 | tetraploid | cold | France | Rhônes-Alpes | 2152 | N |
| 37_2_2 | tetraploid | cold | Italy | Val d'Aosta | 2115 | N |
| 37_3_2 | tetraploid | cold | Italy | Val d'Aosta | 2115 | N |
| 37_4_1 | tetraploid | cold | Italy | Val d'Aosta | 2115 | Y |
| 40_1_2 | tetraploid | cold | Switzerland | Wallis | 1860 | N |
| 40_2_2 | tetraploid | cold | Switzerland | Wallis | 1860 | N |
| 40_3_1 | tetraploid | cold | Switzerland | Wallis | 1860 | Y |
| 40_4_2 | tetraploid | cold | Switzerland | Wallis | 1860 | N |
| 41_2_2 | tetraploid | cold | Italy | Val d'Aosta | 2174 | Y |
| 42_1_1 | tetraploid | cold | Switzerland | Wallis | 1789 | N |
| 42_2_3 | tetraploid | cold | Switzerland | Wallis | 1789 | N |
| 42_3_2 | tetraploid | cold | Switzerland | Wallis | 1789 | Y |
| 42_4_2 | tetraploid | cold | Switzerland | Wallis | 1789 | N |
| 42_4_3 | tetraploid | cold | Switzerland | Wallis | 1789 | N |
| 45_1_1 | tetraploid | cold | Switzerland | Wallis | 2400 | N |
| 45_1_2 | tetraploid | cold | Switzerland | Wallis | 2400 | Y |
| 45_2_1 | tetraploid | cold | Switzerland | Wallis | 2400 | N |
| 45_4_1 | tetraploid | cold | Switzerland | Wallis | 2400 | N |
| 47_1_2 | tetraploid | cold | Switzerland | Graubünden | 2211 | N |
| 47_1_3 | tetraploid | cold | Switzerland | Graubünden | 2211 | N |
| 47_2_2 | tetraploid | cold | Switzerland | Graubünden | 2211 | Y |
| 47_2_3 | tetraploid | cold | Switzerland | Graubünden | 2211 | N |
| 47_4_1 | tetraploid | cold | Switzerland | Graubünden | 2211 | N |
| 48_3_2 | tetraploid | cold | Switzerland | Graubünden | 2262 | N |
| 48_4_2 | tetraploid | cold | Switzerland | Graubünden | 2262 | Y |
| 50_2_1 | tetraploid | cold | Switzerland | Graubünden | 2322 | Y |
| 50_2_2 | tetraploid | cold | Switzerland | Graubünden | 2322 | N |
| 50_3_3 | tetraploid | cold | Switzerland | Graubünden | 2322 | N |
| 50_4_1 | tetraploid | cold | Switzerland | Graubünden | 2322 | N |
| 50_4_3 | tetraploid | cold | Switzerland | Graubünden | 2322 | N |
| 53_2_2 | tetraploid | cold | Switzerland | Graubünden | 2456 | N |
| 53_2_3 | tetraploid | cold | Switzerland | Graubünden | 2456 | N |
| 53_3_1 | tetraploid | cold | Switzerland | Graubünden | 2456 | Y |
| 53_3_3 | tetraploid | cold | Switzerland | Graubünden | 2456 | N |
| 54_1_2 | tetraploid | cold | Italy | Lombardia | 2303 | N |
| 54_2_2 | tetraploid | cold | Italy | Lombardia | 2303 | Y |
| 54_3_3 | tetraploid | cold | Italy | Lombardia | 2303 | N |
| 54_4_2 | tetraploid | cold | Italy | Lombardia | 2303 | N |
| 54_4_3 | tetraploid | cold | Italy | Lombardia | 2303 | N |
| 55_3_1 | tetraploid | cold | Austria | Tirol | 2557 | Y |
| 58_1_2 | tetraploid | cold | Italy | Trentino Alto Adige/ Südtirol | 2117 | N |
| 58_1_3 | tetraploid | cold | Italy | Trentino Alto Adige/ Südtirol | 2117 | Y |
| 58_4_3 | tetraploid | cold | Italy | Trentino Alto Adige/ Südtirol | 2117 | N |
| 66_1_1 | tetraploid | cold | Italy | Trentino Alto Adige/ Südtirol | 2101 | N |
| 66_2_2 | tetraploid | cold | Italy | Trentino Alto Adige/ Südtirol | 2101 | Y |
| 66_2_3 | tetraploid | cold | Italy | Trentino Alto Adige/ Südtirol | 2101 | N |
| 74_2_2 | tetraploid | cold | Austria | Osttirol | 2117 | N |
| 74_4_2 | tetraploid | cold | Austria | Osttirol | 2117 | N |
| 79_3_1 | tetraploid | cold | Switzerland | Graubünden | 2280 | N |
| 79_3_3 | tetraploid | cold | Switzerland | Graubünden | 2280 | Y |
| 82_3_3 | tetraploid | cold | Italy | Lombardia | 2500 | Y |
| 83_3_1 | tetraploid | cold | Austria | Osttirol | 2271 | Y |
| 84_1_2 | tetraploid | cold | Austria | Kärnten | 2236 | N |
| 84_4_2 | tetraploid | cold | Austria | Kärnten | 2236 | Y |
| 85_3_2 | tetraploid | cold | Austria | Kärnten | 2184 | N |
| 85_3_3 | tetraploid | cold | Austria | Kärnten | 2184 | N |
| 88_1_1 | tetraploid | cold | Switzerland | Graubünden | 2300 | N |
| 88_1_2 | tetraploid | cold | Switzerland | Graubünden | 2300 | N |
| 88_2_3 | tetraploid | cold | Switzerland | Graubünden | 2300 | N |
| 90_2_3 | tetraploid | cold | Switzerland | Wallis | 2477 | Y |
| 92_2_1 | tetraploid | cold | Switzerland | Graubünden | 2260 | N |
| 92_4_1 | tetraploid | cold | Switzerland | Graubünden | 2260 | N |
| 93_2_1 | tetraploid | cold | Switzerland | Wallis | 2405 | N |
| 93_2_3 | tetraploid | cold | Switzerland | Wallis | 2405 | N |
| 93_3_3 | tetraploid | cold | Switzerland | Wallis | 2405 | N |
| 96_1_3 | tetraploid | cold | France | Provence-Alpes-Côte d'Azur | 2300 | N |
| 96_2_3 | tetraploid | cold | France | Provence-Alpes-Côte d'Azur | 2300 | Y |
| 96_3_2 | tetraploid | cold | France | Provence-Alpes-Côte d'Azur | 2300 | N |
| 96_4_2 | tetraploid | cold | France | Provence-Alpes-Côte d'Azur | 2300 | N |
| 96_4_3 | tetraploid | cold | France | Provence-Alpes-Côte d'Azur | 2300 | N |
| 103_1_2 | tetraploid | cold | Italy | Lombardia | 2290 | N |
| 106_2_1 | tetraploid | cold | Italy | Trentino Alto Adige/ Südtirol | 2142 | N |
| 106_2_2 | tetraploid | cold | Italy | Trentino Alto Adige/ Südtirol | 2142 | N |
| 106_2_3 | tetraploid | cold | Italy | Trentino Alto Adige/ Südtirol | 2142 | Y |
| 106_4_3 | tetraploid | cold | Italy | Trentino Alto Adige/ Südtirol | 2142 | N |
| 108_3_2 | tetraploid | cold | Switzerland | Graubünden | 2171 | N |
| 108_3_3 | tetraploid | cold | Switzerland | Graubünden | 2171 | Y |
| 111_1_1 | tetraploid | cold | France | Provence-Alpes-Côte d'Azur | 2243 | N |
| 111_1_2 | tetraploid | cold | France | Provence-Alpes-Côte d'Azur | 2243 | N |
| 111_2_3 | tetraploid | cold | France | Provence-Alpes-Côte d'Azur | 2243 | Y |

**Table S2.** Reproductive modes (a) and special cases of assumed reproduction modes (b) for diploid and tetraploid *Ranunculus kuepferi* plants under temperature treatments. The FCSS data for embryo and endosperm ploidy in single seeds presented in (b) were excluded from further analysis.

|  | Genome contribution to embryo/endosperm | Embryo: endosperm | Peak index | Path | Number of observations (seeds, ssFCSS) | | |
| --- | --- | --- | --- | --- | --- | --- | --- |
|  | Egg cell + sperm nucleus/fused polar nuclei + sperm nucleus (nuclei) |  |  |  | Cold | Warm | Total |
| a) Reproduction mode | | | | | | | |
| Diploid plants | | | | | | | |
| Sexual | 1Cx(m) + 1Cx(p)/2Cx(m) + 1Cx(p) | 2:3 | 1.5 | A | 206 | 359 | 565 |
| Apomictic | 2Cx(m) + 0Cx(p)/4Cx(m) + 0Cx(p) | 2:4 | 2 | B | 1 | 6 | 7 |
|  | 2Cx(m) + 0Cx(p)/4Cx(m) + 1Cx(p) | 2:5 | 2.5 | C | 0 | 2 | 2 |
|  | 2Cx(m) + 0Cx(p)/4Cx(m) + 2Cx(p) | 2:6 | 3 | D | 2 | 0 | 2 |
| B_III_ hybrid | 2Cx(m) + 1Cx(p)/4Cx(m) + 1Cx(p) | 3:5 | 1.67 | E | 0 | 7 | 7 |
|  | 2Cx(m) + 2Cx(p)/4Cx(m) + 2Cx(p) | 4:6 | 1.5 | F | 0 | 1 | 1 |
|  | 2Cx(m) + 2Cx(p)/(4Cx(m))*2 + 2Cx(p) | 4:10 | 2.5 | G | 0 | 8 | 8 |
| Tetraploid plants | | | | | | | |
| Sexual | 2Cx(m) + 2Cx(p)/4Cx(m) + 2Cx(p) | 4:6 | 1.5 | H | 6 | 0 | 6 |
| Apomictic | 4Cx(m) + 0Cx(p)/8Cx(m) + 0Cx(p) | 4:8 | 2 | I | 2 | 0 | 2 |
|  | 4Cx(m) + 0Cx(p)/8Cx(m) + 1Cx(p) | 4:9 | 2.25 | J | 3 | 5 | 8 |
|  | 4Cx(m) + 0Cx(p)/8Cx(m) + 2Cx(p) | 4:10 | 2.5 | K | 52 | 1 | 53 |
|  | 4Cx(m) + 0Cx(p)/8Cx(m) + 3Cx(p) | 4:11 | 2.75 | L | 8 | 0 | 8 |
|  | 4Cx(m) + 0Cx(p)/8Cx(m) + 4Cx(p) | 4:12 | 3 | M | 11 | 0 | 11 |
|  | 4Cx(m) + 0Cx(p)/8Cx(m) + 6Cx(p) | 4:14 | 3.5 | N | 8 | 0 | 8 |
|  |  |  |  |  |  |  |  |
| b) Assumed reproduction mode | | | | | | | |
| Diploid plants | | | | | | | |
| Apospory with polyspermy | 2Cx(m) + 0Cx(p)/4Cx(m) + 3Cx(p) | 2:7 | 3.5 |  | 2 | 1 | 3 |
| Apospory with polyspermy | 2Cx(m) + 1Cx(p)/4Cx(m) + 3Cx(p) | 3:7 | 2.33 |  | 1 | 1 | 2 |
| Apospory with polyspermy | 2Cx(m) + 1Cx(p)/4Cx(m) + 4Cx(p) | 3:8 | 2.67 |  | 0 | 2 | 2 |
| Apospory with polyspermy | 2Cx(m) +2Cx(p)/4Cx(m) + 5Cx(p) | 4:9 | 2.25 |  | 0 | 2 | 2 |
| Apospory with endosperm endopolyploidy & polyspermy | 2Cx(m) + 2Cx(p)/(4Cx(m))*2 + 8Cx(p) | 4:16 | 4 |  | 0 | 1 | 1 |
|  |  |  |  |  |  |  |  |
| Tetraploid plants | | | | | | | |
| Apospory with endopolyploidy | 4Cx(m) + 0Cx(p)/(8Cx(m))*2 + 2Cx(p) | 4:18 | 1:4.5 |  | 2 | 0 | 2 |
| Hypertetraploid aneuploidy with autonomous endosperm | 5Cx(m) + 0Cx(p)/10Cx(m) + 0Cx(p) | 5:10 | 2 |  | 1 | 0 | 1 |

Cx :ploidy based on DNA content; m: maternal genome contribution; p: paternal genome contribution.

Table S3. Vegetative growth data for the measured morphological traits, seed yield data and reproduction mode of the seeds in diploid and tetraploid Ranunculus kuepferi plants under temperature treatments (Supplementary excel file).


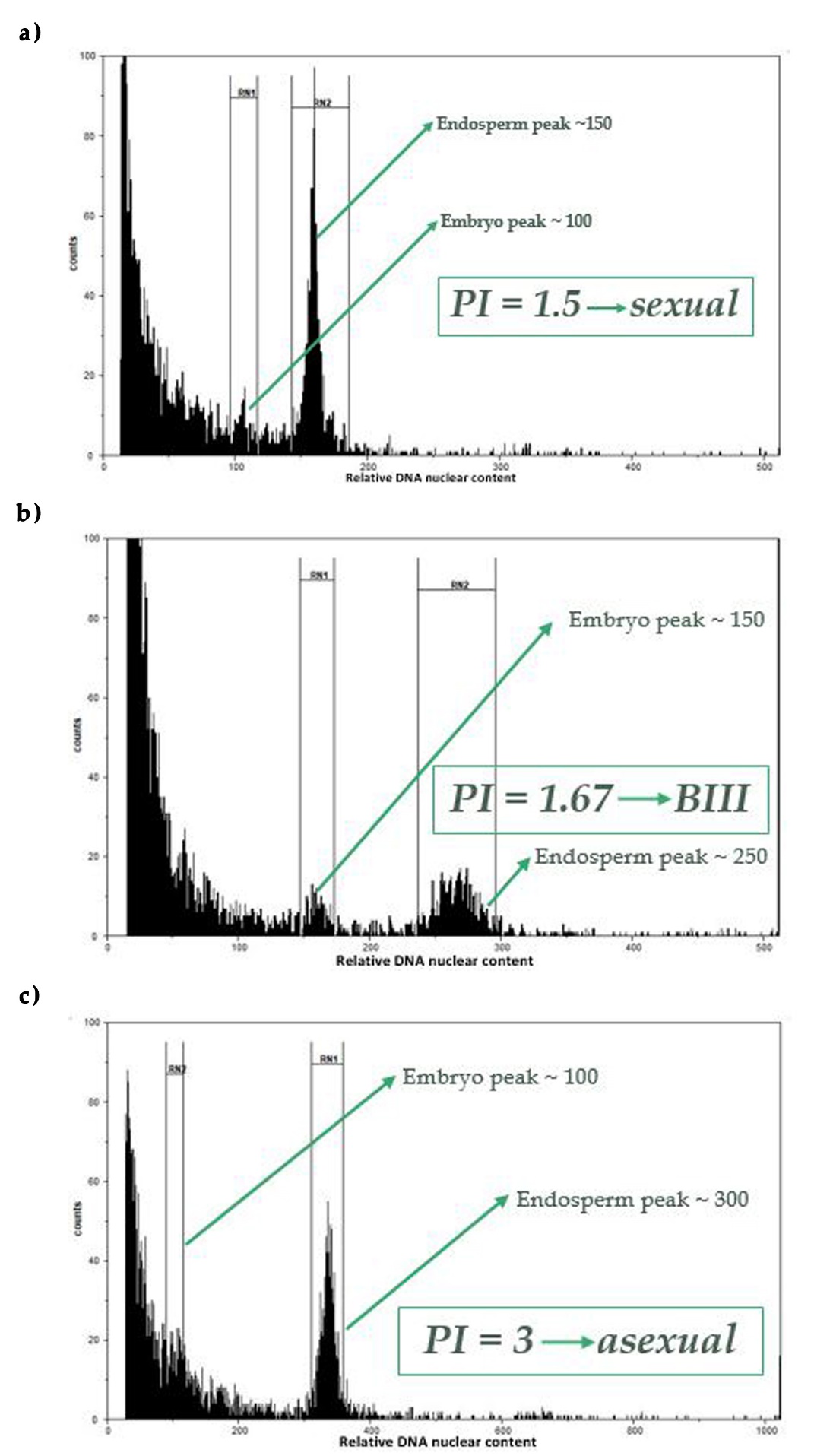


**Figure S1.** Representative histograms of sexual (a), BIII hybrid (b) and apomictic seeds (c) of *Ranunculus kuepferi*. All histograms refer to seeds of diploid mother plants.


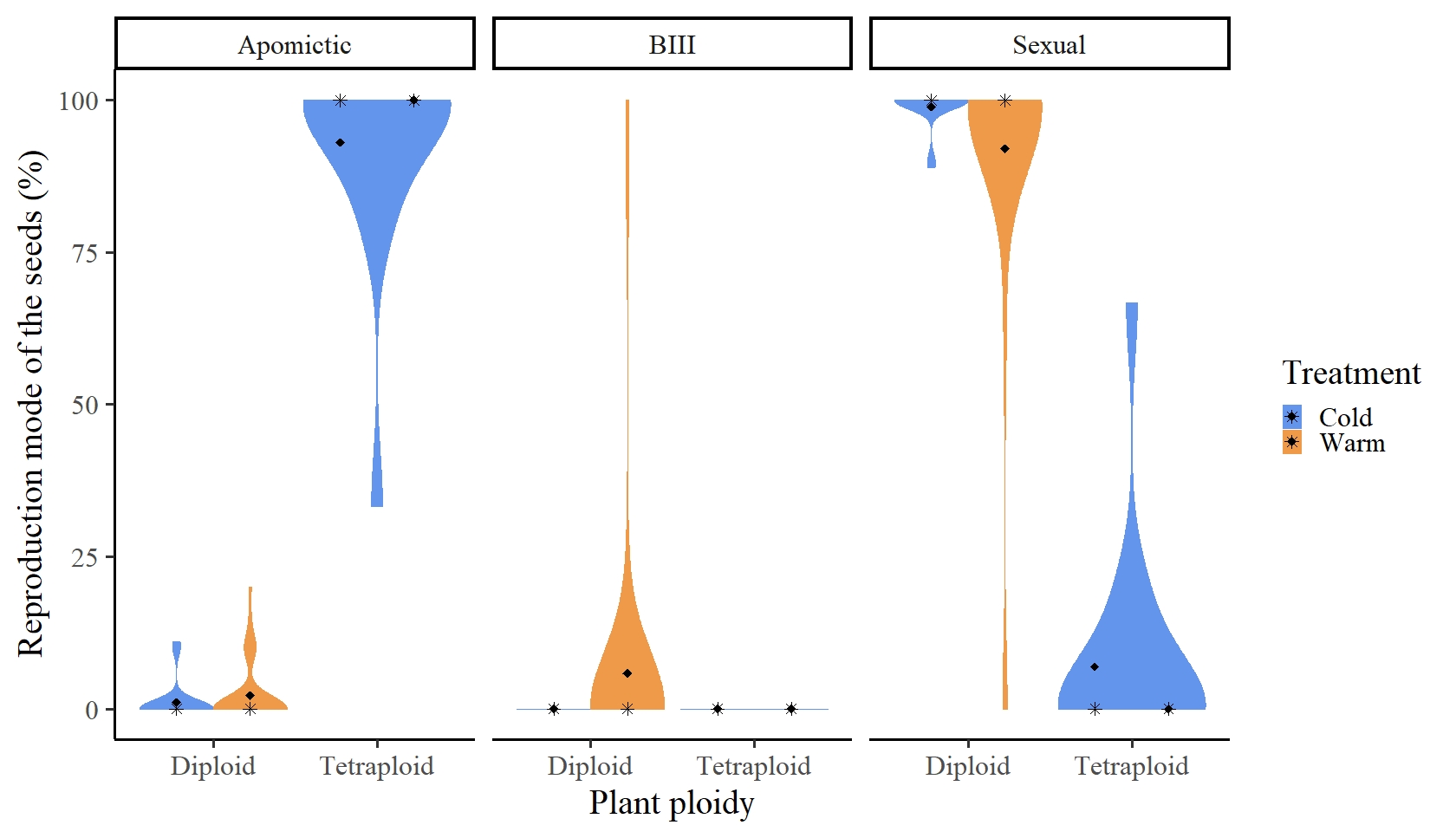


**Figure S2.** Influence of the temperature treatments on the mode of reproduction in diploid and tetraploid *Ranunculus kuepferi* plants. Violin plots show the percentages of apomictic seeds, BIII hybrids (partially apomictic seeds) and sexual seeds produced by plants under the cold and warm treatment.

**Methods S1.** Methylation-sensitive amplified fragment length polymorphisms (MS-AFLPs or MSAPs): Lab protocol and fragments scoring pipeline [70].

DNA from the leaf material was isolated using the Qiagen DNeasy Plant Mini Kit, with a slightly modified protocol. At the second step, 360 μl AP1 Buffer and 40 μl PVP 2.6% were added and incubation time for the elution was prolonged to 30 min. The extracted samples of 100 individuals for each treatment year were screened according to a slightly modified protocol of Paun *et al.* [72]. Restriction and ligation were carried out in two parallel reactions, each one with a different restriction enzyme. The restriction enzymes, which were used, are MspI & HpaII. They are methylation sensitive restriction enzymes, i.e. isoschizomers, that recognize the same DNA sequence (CCGG), but differ in the sensitivity regarding the methylation state of C, and used as the frequent cutters, while EcoRI is used as the rare cutter. Ligation products were subjected to pre-selective amplification, whereupon selective amplification was performed with a set of three primer combinations with three selective nucleotides to each primer, used before for an AFLP analysis on the species [62]. Ligation, pre-selective and selective amplification products went through a quality and quantity check on a 1.5% agarose gel and diluted 10-fold dilution prior to pre-selective, selective amplification and fragment analyses, respectively. The final selective-PCR products were prepared with GeneScan ROX 500 (Thermo Fisher Scientific, Waltham, MA, USA) as the internal size standard and fragment analyzed on the ABI Prism 3700/3730 (Applied Biosystems, Waltham, MA, USA) capillary sequencer.

The technical reproducibility of resulting electropherograms was checked by replicating 100% of accessions, i.e. duplicates were produced for every sample used throughout the MSAP protocol steps, to minimize the false positive fragment peaks. We transformed electropherograms of raw data into a binary dominant-marker matrix. Peak Scanner2 was used to determine the height, width & the area of all peaks. The output of the Peak Scanner2 was then imported to RawGeno 2.0-1 [73] to proceed with the binning of detected peaks, the analysis of replication and the filtering of samples of low quality. RawGeno handles a single dye color at a time, so presence/absence of fragments binary matrices were obtained for each of the three dyes (Blue; FAM, Green; HEX, Yellow; NED) and then they were merged in a final binary matrix. Fragments between 50 and 600 base pairs were scored. In order to optimize the fragment detection and minimize the risk of false positives, a run of RawGeno with an R script [73] was performed, which checked stepwise (~5760 steps) the binning and filtering parameters. Going through the resulting table, the optimal combination of the parameters was chosen for each dye and the respective binary matrices were produced. The selection of parameters represent a balance between quality measures, e.g. the error rate and bin reproducibility, and informativity, measured with the data polymorphism.

The merged binary matrix of optimized dataset for each treatment year was dealt with MSAP_calc script in R [74], to distinguish the four possible methylation conditions, using the ‘Mixed Scoring 2’ approach for scoring the following conditions: I) no methylation (both MspI and HpaII cut the restriction site), II) holo- or hemi-methylation of internal cytosine (^HMe^CG or ^Me^CG; MspI cuts the restriction site), III) hemimethylation of external cytosine (^HMe^CCG; HpaII cuts the restriction site) and IV) holomethylation of external cytosine or of both cytosines or hemi-methylation of both cytosines or mutations (none of them cuts the restriction site). In ‘Mixed Scoring 2’ condition I was scored as 100 (non-methylated), condition II as 010 (internally-methylated), condition III as 001 (externally methylated), and condition IV as ‘000’ and refers to a non-distinguishable situation, e.g. an ambiguous methylation or a mutation status. Condition IV was, therefore, excluded from further statistical analyses.

| 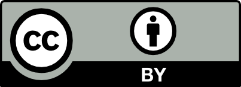 | © 2020 by the authors. Submitted for possible open access publication under the terms and conditions of the Creative Commons Attribution (CC BY) license (http://creativecommons.org/licenses/by/4.0/). |
| --- | --- |
